# Phen­yl(3-methyl-1-phenyl­sulfonyl-1*H*-indol-2-yl)methanone

**DOI:** 10.1107/S1600536811014826

**Published:** 2011-04-29

**Authors:** S. Ranjith, A. SubbiahPandi, V. Dhayalan, A. K. MohanaKrishnan

**Affiliations:** a Department of Physics, Presidency College (Autonomous), Chennai 600 005, India; b Department of Organic Chemistry, University of Madras, Guindy Campus, Chennai 600 025, India

## Abstract

In the title compound, C_22_H_17_NO_3_S, the N atom of the indole ring system deviates by 0.031 (3) Å from a least-squares plane fitted through all nine non-H ring atoms. The geometry around the S atom can be described as distorted tetra­hedral. As a result of the electron-withdrawing character of the phenyl­sulfonyl groups, the N—C*sp* ^2^ bond lengths are longer than the typical mean value for N atoms with a planar configuration.

## Related literature

For background to the biological activity of indole-containing compounds, see: Ma *et al.* (2001 ▶). For a related structure, see: Ranjith *et al.* (2011 ▶). For discussion of bond angles around N atoms, see: Beddoes *et al.* (1986 ▶).
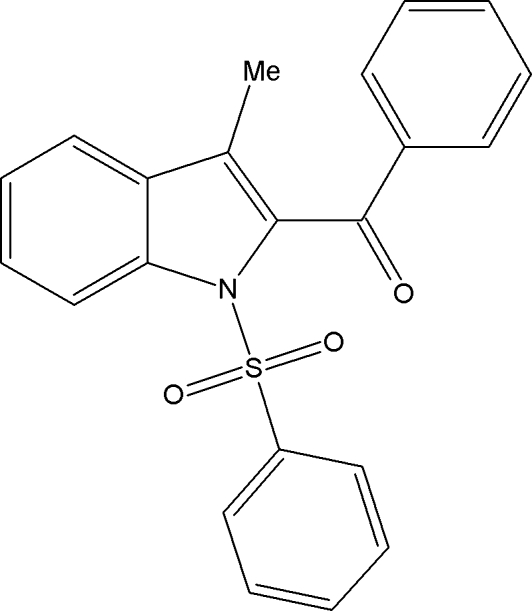


## Experimental

### 

#### Crystal data

C_22_H_17_NO_3_S
*M* *_r_* = 375.43
Monoclinic, 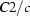

*a* = 22.8120 (7) Å
*b* = 10.5199 (4) Å
*c* = 16.1346 (6) Å
β = 103.926 (1)°
*V* = 3758.2 (2) Å^3^
*Z* = 8
Mo *K*α radiation
μ = 0.19 mm^−1^
*T* = 293 K
0.25 × 0.22 × 0.19 mm

#### Data collection

Bruker APEXII CCD area-detector diffractometer
Absorption correction: multi-scan (*SADABS*; Sheldrick, 1996 ▶) *T*_min_ = 0.953, *T*_max_ = 0.964
24166 measured reflections
5682 independent reflections
3487 reflections with *I* > 2σ(*I*)
*R*_int_ = 0.031

#### Refinement

*R*[*F* ^2^ > 2σ(*F* ^2^)] = 0.049
*wR*(*F* ^2^) = 0.145
*S* = 1.02
5682 reflections
245 parameters
H-atom parameters constrained
Δρ_max_ = 0.23 e Å^−3^
Δρ_min_ = −0.36 e Å^−3^

### 

Data collection: *APEX2* (Bruker, 2007 ▶); cell refinement: *SAINT* (Bruker, 2007 ▶); data reduction: *SAINT*; program(s) used to solve structure: *SHELXTL* (Sheldrick, 2008 ▶); program(s) used to refine structure: *SHELXTL*; molecular graphics: *ORTEP-3* (Farrugia, 1997 ▶); software used to prepare material for publication: *SHELXTL* and *PLATON* (Spek, 2009 ▶).

## Supplementary Material

Crystal structure: contains datablocks global, I. DOI: 10.1107/S1600536811014826/nk2097sup1.cif

Structure factors: contains datablocks I. DOI: 10.1107/S1600536811014826/nk2097Isup2.hkl

Supplementary material file. DOI: 10.1107/S1600536811014826/nk2097Isup3.cml

Additional supplementary materials:  crystallographic information; 3D view; checkCIF report

## Figures and Tables

**Table 1:** Hydrogen-bond geometry (Å, °)

*D*—H⋯*A*	*D*—H	H⋯*A*	*D*⋯*A*	*D*—H⋯*A*
C2—H2⋯O1	0.93	2.45	3.025 (2)	120
C15—H15⋯N1	0.93	2.61	3.254 (2)	127
C21—H21⋯O1	0.93	2.53	2.904 (2)	104
C21—H21⋯O2^i^	0.93	2.29	3.127 (2)	149

Symmetry code: (i) 
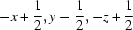
.

**Fig. 1.**
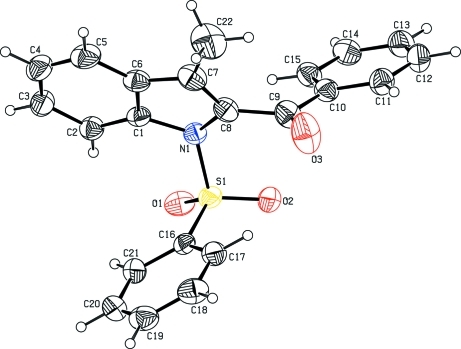
The structure of showing the atom-numbering scheme and intramolecular hydrogen bond. Displacement ellipsoids are drawn at the 30% probability level.

## supplementary crystallographic information

### Comment

The indole ring system is present in a number of natural products, many of which are found to possess anticancer, antimalarial and antihypertensive activities (Ma *et al.*, 2001). Sulfonamide derivates are well known drugs and are used to control diseases caused by bacterial infections. Against this background and in order to obtain detailed information on molecular conformations in the solid state, X-ray study of the title compound was carried out.

X-Ray analysis confirms the molecular structure and atom connectivity as illustrated in Fig. 1. The indole ring system is essentially planar with a mean deviation from a plane defined by the nine non-H ring atoms of 0.031 (3) Å for N. The sum of bond angles around N1 [348.4 (12)°] of the pyrrole ring is in accordance with *sp*^3^ hybridization (Beddoes *et al.*, 1986). The indole ring system makes dihedral angles of 61.7 (8) and 77.7 (8)°, respectively, with the benzene and phenyl rings.

The S—O, S—C, and S—N distances are 1.425 (2), 1.748 (2) and 1.670 (1) Å, respectively and these values are comparable as observed in similar structures (Ranjith *et al.*, 2011). As a result of the electron-withdrawing character of the phenylsulfonyl groups, the N—C*sp*^2^ bond lengths, *viz*. N1—C1 [1.420 (2) Å] and N1—C8 [1.422 (2) Å] are longer than the mean value of 1.355 (1) Å reported for N atoms with planar configurations (Ranjith *et al.*, 2011).

### Experimental

To a solution of *N*-(2-acetylphenyl)benzenesulfonamide (0.4 g, 1.45 mmol) in dry CH_3_CN (20 ml), K_2_CO_3_ (0.6 g, 4.34 mmol), 2-bromo-1-phenylethanone (0.34 g, 1.70 mmol) were added. The reaction mixture was stirred at room temperature for 6 h under N_2_ atmosphere. The solvent was removed and the residue was quenched with ice–water (50 ml), extracted with chloroform (3 × 10 ml) and dried (Na_2_SO_4_). Removal of solvent followed by the residue was dissolved in CH_3_CN (20 ml), Concentrated HCl (3 ml) was added. The reaction mixture was then refluxed for 2 h. It was then poured over ice–water (50 ml), extracted with CHCl_3_ (3 × 10 ml) and dried (Na_2_SO_4_). Single crystals suitable for X-ray diffraction were obtained by slow evaporation of a solution of the title compound in methanol at room temperature.

### Refinement

All H atoms were fixed geometrically and allowed to ride on their parent C atoms, with C—H distances fixed in the range 0.93–0.97 Å with *U*_iso_(H) = 1.5*U*_eq_(C) for methyl H 1.2*U*_eq_(C) for other H atoms.

### Crystal data

**Table:**

C_22_H_17_NO_3_S	*F*(000) = 1568
*M**_r_* = 375.43	*D*_x_ = 1.327 Mg m^−^^3^
Monoclinic, *C*2/*c*	Mo *K*α radiation, λ = 0.71073 Å
Hall symbol: -C 2yc	Cell parameters from 5682 reflections
*a* = 22.8120 (7) Å	θ = 2.1–30.4°
*b* = 10.5199 (4) Å	µ = 0.19 mm^−^^1^
*c* = 16.1346 (6) Å	*T* = 293 K
β = 103.926 (1)°	Block, white
*V* = 3758.2 (2) Å^3^	0.25 × 0.22 × 0.19 mm
*Z* = 8	

### Data collection

**Table:**

Bruker APEXII CCD area-detector diffractometer	5682 independent reflections
Radiation source: fine-focus sealed tube	3487 reflections with *I* > 2σ(*I*)
graphite	*R*_int_ = 0.031
ω and φ scans	θ_max_ = 30.4°, θ_min_ = 2.1°
Absorption correction: multi-scan (*SADABS*; Sheldrick, 1996)	*h* = −32→25
*T*_min_ = 0.953, *T*_max_ = 0.964	*k* = −11→14
24166 measured reflections	*l* = −22→23

### Refinement

**Table:**

Refinement on *F*^2^	Primary atom site location: structure-invariant direct methods
Least-squares matrix: full	Secondary atom site location: difference Fourier map
*R*[*F*^2^ > 2σ(*F*^2^)] = 0.049	Hydrogen site location: inferred from neighbouring sites
*wR*(*F*^2^) = 0.145	H-atom parameters constrained
*S* = 1.02	*w* = 1/[σ^2^(*F*_o_^2^) + (0.069*P*)^2^ + 1.0112*P*] where *P* = (*F*_o_^2^ + 2*F*_c_^2^)/3
5682 reflections	(Δ/σ)_max_ = 0.001
245 parameters	Δρ_max_ = 0.23 e Å^−^^3^
0 restraints	Δρ_min_ = −0.36 e Å^−^^3^

### Special details

**Table:**

Geometry. All e.s.d.'s (except the e.s.d. in the dihedral angle between two l.s. planes) are estimated using the full covariance matrix. The cell e.s.d.'s are taken into account individually in the estimation of e.s.d.'s in distances, angles and torsion angles; correlations between e.s.d.'s in cell parameters are only used when they are defined by crystal symmetry. An approximate (isotropic) treatment of cell e.s.d.'s is used for estimating e.s.d.'s involving l.s. planes.
Refinement. Refinement of *F*^2^ against ALL reflections. The weighted *R*-factor *wR* and goodness of fit *S* are based on *F*^2^, conventional *R*-factors *R* are based on *F*, with *F* set to zero for negative *F*^2^. The threshold expression of *F*^2^ > σ(*F*^2^) is used only for calculating *R*-factors(gt) *etc*. and is not relevant to the choice of reflections for refinement. *R*-factors based on *F*^2^ are statistically about twice as large as those based on *F*, and *R*-factors based on ALL data will be even larger.

### Fractional atomic coordinates and isotropic or equivalent isotropic displacement parameters (Å^2^)

**Table:**

	*x*	*y*	*z*	*U*_iso_*/*U*_eq_	
C5	0.02854 (9)	0.3172 (2)	0.15082 (14)	0.0761 (6)	
H5	−0.0129	0.3160	0.1459	0.091*	
C4	0.06108 (10)	0.2069 (2)	0.16354 (14)	0.0753 (6)	
H4	0.0415	0.1303	0.1674	0.090*	
C3	0.12244 (9)	0.20748 (18)	0.17070 (12)	0.0672 (5)	
H3	0.1435	0.1310	0.1792	0.081*	
C2	0.15354 (8)	0.31829 (16)	0.16568 (11)	0.0566 (4)	
H2	0.1951	0.3180	0.1711	0.068*	
C1	0.12076 (7)	0.42995 (16)	0.15223 (10)	0.0487 (3)	
C6	0.05851 (7)	0.43214 (19)	0.14524 (11)	0.0593 (4)	
C7	0.03865 (8)	0.5620 (2)	0.13190 (13)	0.0688 (5)	
C8	0.08715 (7)	0.63472 (17)	0.13080 (11)	0.0581 (4)	
C22	−0.02630 (10)	0.6044 (3)	0.1168 (2)	0.1116 (10)	
H22A	−0.0304	0.6886	0.0930	0.167*	
H22B	−0.0382	0.6047	0.1700	0.167*	
H22C	−0.0517	0.5470	0.0777	0.167*	
C9	0.08567 (9)	0.77369 (19)	0.11124 (13)	0.0692 (5)	
C10	0.09789 (8)	0.82165 (16)	0.03063 (11)	0.0579 (4)	
C11	0.07967 (9)	0.94417 (18)	0.00483 (13)	0.0683 (5)	
H11	0.0613	0.9946	0.0387	0.082*	
C12	0.08861 (10)	0.9915 (2)	−0.07082 (15)	0.0775 (6)	
H12	0.0757	1.0731	−0.0883	0.093*	
C13	0.11653 (10)	0.9185 (2)	−0.12028 (14)	0.0763 (6)	
H13	0.1230	0.9514	−0.1709	0.092*	
C14	0.13492 (9)	0.7980 (2)	−0.09585 (13)	0.0718 (5)	
H14	0.1541	0.7491	−0.1296	0.086*	
C15	0.12516 (8)	0.74826 (18)	−0.02105 (12)	0.0631 (4)	
H15	0.1369	0.6653	−0.0053	0.076*	
C16	0.20700 (7)	0.60409 (14)	0.30154 (10)	0.0485 (4)	
C17	0.17865 (8)	0.69870 (17)	0.33647 (12)	0.0621 (4)	
H17	0.1590	0.7650	0.3028	0.074*	
C18	0.17995 (9)	0.6930 (2)	0.42209 (13)	0.0748 (6)	
H18	0.1609	0.7560	0.4465	0.090*	
C19	0.20885 (10)	0.5959 (2)	0.47163 (13)	0.0754 (6)	
H19	0.2095	0.5933	0.5295	0.090*	
C20	0.23697 (10)	0.5021 (2)	0.43652 (13)	0.0750 (6)	
H20	0.2566	0.4362	0.4706	0.090*	
C21	0.23613 (8)	0.50521 (17)	0.35097 (12)	0.0621 (4)	
H21	0.2549	0.4416	0.3268	0.075*	
N1	0.13917 (5)	0.55634 (13)	0.14070 (8)	0.0504 (3)	
O1	0.24984 (5)	0.51956 (13)	0.17690 (8)	0.0667 (4)	
O2	0.20878 (6)	0.73760 (13)	0.16764 (9)	0.0764 (4)	
O3	0.06769 (10)	0.84532 (17)	0.15969 (12)	0.1136 (6)	
S1	0.207048 (18)	0.60904 (4)	0.19323 (3)	0.05342 (14)	

### Atomic displacement parameters (Å^2^)

**Table:**

	*U*^11^	*U*^22^	*U*^33^	*U*^12^	*U*^13^	*U*^23^
C5	0.0493 (10)	0.0991 (16)	0.0790 (14)	−0.0230 (10)	0.0134 (9)	0.0018 (11)
C4	0.0763 (13)	0.0729 (13)	0.0756 (13)	−0.0247 (11)	0.0161 (10)	0.0001 (10)
C3	0.0762 (12)	0.0569 (10)	0.0683 (12)	−0.0079 (9)	0.0169 (10)	−0.0009 (9)
C2	0.0516 (9)	0.0594 (10)	0.0589 (10)	−0.0024 (7)	0.0134 (8)	−0.0002 (8)
C1	0.0422 (7)	0.0588 (9)	0.0442 (8)	−0.0075 (6)	0.0089 (6)	−0.0027 (7)
C6	0.0428 (8)	0.0773 (12)	0.0565 (10)	−0.0052 (8)	0.0093 (7)	0.0003 (9)
C7	0.0487 (9)	0.0880 (14)	0.0707 (12)	0.0101 (9)	0.0164 (9)	0.0053 (10)
C8	0.0535 (9)	0.0649 (10)	0.0564 (10)	0.0102 (8)	0.0142 (8)	0.0030 (8)
C22	0.0530 (12)	0.135 (2)	0.150 (3)	0.0246 (13)	0.0303 (14)	0.0199 (19)
C9	0.0755 (12)	0.0685 (12)	0.0652 (11)	0.0184 (10)	0.0200 (10)	−0.0017 (9)
C10	0.0531 (9)	0.0575 (10)	0.0594 (10)	0.0056 (7)	0.0063 (8)	−0.0012 (8)
C11	0.0672 (11)	0.0567 (10)	0.0796 (13)	0.0070 (9)	0.0145 (10)	−0.0024 (9)
C12	0.0796 (14)	0.0596 (12)	0.0886 (15)	−0.0007 (10)	0.0110 (12)	0.0145 (11)
C13	0.0770 (13)	0.0802 (14)	0.0694 (12)	−0.0102 (11)	0.0130 (10)	0.0121 (11)
C14	0.0691 (12)	0.0820 (14)	0.0641 (12)	0.0036 (10)	0.0158 (10)	−0.0001 (10)
C15	0.0643 (10)	0.0613 (10)	0.0604 (10)	0.0116 (8)	0.0089 (8)	0.0019 (8)
C16	0.0431 (7)	0.0449 (8)	0.0552 (9)	−0.0064 (6)	0.0075 (7)	−0.0017 (7)
C17	0.0603 (10)	0.0564 (10)	0.0655 (11)	0.0067 (8)	0.0072 (8)	−0.0034 (8)
C18	0.0669 (12)	0.0868 (14)	0.0714 (13)	0.0021 (10)	0.0183 (10)	−0.0201 (11)
C19	0.0761 (13)	0.0931 (16)	0.0565 (11)	−0.0167 (11)	0.0150 (10)	−0.0039 (11)
C20	0.0831 (14)	0.0692 (13)	0.0645 (12)	−0.0053 (10)	0.0020 (10)	0.0134 (10)
C21	0.0675 (11)	0.0514 (10)	0.0640 (11)	0.0036 (8)	0.0093 (9)	0.0005 (8)
N1	0.0422 (7)	0.0539 (8)	0.0537 (7)	−0.0025 (5)	0.0088 (6)	0.0010 (6)
O1	0.0425 (6)	0.0876 (9)	0.0726 (8)	−0.0042 (6)	0.0193 (6)	−0.0121 (7)
O2	0.0855 (9)	0.0646 (8)	0.0716 (8)	−0.0255 (7)	0.0043 (7)	0.0147 (6)
O3	0.1770 (18)	0.0842 (11)	0.0986 (12)	0.0421 (12)	0.0703 (13)	0.0065 (9)
S1	0.0469 (2)	0.0572 (3)	0.0552 (3)	−0.01117 (17)	0.01038 (17)	0.00133 (18)

### Geometric parameters (Å, °)

**Table:**

C5—C4	1.366 (3)	C11—H11	0.9300
C5—C6	1.403 (3)	C12—C13	1.370 (3)
C5—H5	0.9300	C12—H12	0.9300
C4—C3	1.377 (3)	C13—C14	1.364 (3)
C4—H4	0.9300	C13—H13	0.9300
C3—C2	1.377 (2)	C14—C15	1.382 (3)
C3—H3	0.9300	C14—H14	0.9300
C2—C1	1.381 (2)	C15—H15	0.9300
C2—H2	0.9300	C16—C17	1.379 (2)
C1—C6	1.397 (2)	C16—C21	1.380 (2)
C1—N1	1.420 (2)	C16—S1	1.7485 (17)
C6—C7	1.439 (3)	C17—C18	1.376 (3)
C7—C8	1.348 (3)	C17—H17	0.9300
C7—C22	1.510 (3)	C18—C19	1.365 (3)
C8—N1	1.422 (2)	C18—H18	0.9300
C8—C9	1.494 (3)	C19—C20	1.372 (3)
C22—H22A	0.9600	C19—H19	0.9300
C22—H22B	0.9600	C20—C21	1.376 (3)
C22—H22C	0.9600	C20—H20	0.9300
C9—O3	1.225 (2)	C21—H21	0.9300
C9—C10	1.483 (3)	N1—S1	1.6709 (13)
C10—C11	1.387 (3)	O1—S1	1.4257 (13)
C10—C15	1.389 (2)	O2—S1	1.4174 (13)
C11—C12	1.378 (3)		
			
C4—C5—C6	119.11 (18)	C13—C12—C11	120.15 (19)
C4—C5—H5	120.4	C13—C12—H12	119.9
C6—C5—H5	120.4	C11—C12—H12	119.9
C5—C4—C3	120.89 (19)	C14—C13—C12	120.4 (2)
C5—C4—H4	119.6	C14—C13—H13	119.8
C3—C4—H4	119.6	C12—C13—H13	119.8
C4—C3—C2	121.80 (19)	C13—C14—C15	120.1 (2)
C4—C3—H3	119.1	C13—C14—H14	120.0
C2—C3—H3	119.1	C15—C14—H14	120.0
C3—C2—C1	117.52 (16)	C14—C15—C10	120.25 (18)
C3—C2—H2	121.2	C14—C15—H15	119.9
C1—C2—H2	121.2	C10—C15—H15	119.9
C2—C1—C6	121.86 (16)	C17—C16—C21	121.05 (17)
C2—C1—N1	130.61 (14)	C17—C16—S1	120.01 (13)
C6—C1—N1	107.51 (14)	C21—C16—S1	118.94 (13)
C1—C6—C5	118.82 (18)	C18—C17—C16	118.66 (17)
C1—C6—C7	107.75 (15)	C18—C17—H17	120.7
C5—C6—C7	133.42 (17)	C16—C17—H17	120.7
C8—C7—C6	108.09 (15)	C19—C18—C17	120.76 (19)
C8—C7—C22	127.3 (2)	C19—C18—H18	119.6
C6—C7—C22	124.5 (2)	C17—C18—H18	119.6
C7—C8—N1	109.56 (16)	C18—C19—C20	120.3 (2)
C7—C8—C9	125.49 (17)	C18—C19—H19	119.8
N1—C8—C9	124.53 (15)	C20—C19—H19	119.8
C7—C22—H22A	109.5	C19—C20—C21	120.12 (19)
C7—C22—H22B	109.5	C19—C20—H20	119.9
H22A—C22—H22B	109.5	C21—C20—H20	119.9
C7—C22—H22C	109.5	C20—C21—C16	119.10 (18)
H22A—C22—H22C	109.5	C20—C21—H21	120.5
H22B—C22—H22C	109.5	C16—C21—H21	120.5
O3—C9—C10	120.85 (19)	C1—N1—C8	107.01 (12)
O3—C9—C8	117.45 (19)	C1—N1—S1	120.76 (11)
C10—C9—C8	121.25 (16)	C8—N1—S1	120.73 (12)
C11—C10—C15	118.80 (18)	O2—S1—O1	120.52 (9)
C11—C10—C9	118.25 (17)	O2—S1—N1	104.85 (8)
C15—C10—C9	122.92 (16)	O1—S1—N1	106.22 (7)
C12—C11—C10	120.25 (19)	O2—S1—C16	109.05 (8)
C12—C11—H11	119.9	O1—S1—C16	109.29 (8)
C10—C11—H11	119.9	N1—S1—C16	105.88 (7)
			
C6—C5—C4—C3	−0.1 (3)	C13—C14—C15—C10	−1.5 (3)
C5—C4—C3—C2	0.2 (3)	C11—C10—C15—C14	1.3 (3)
C4—C3—C2—C1	−0.6 (3)	C9—C10—C15—C14	179.47 (18)
C3—C2—C1—C6	1.0 (3)	C21—C16—C17—C18	0.1 (3)
C3—C2—C1—N1	−176.99 (16)	S1—C16—C17—C18	−179.28 (14)
C2—C1—C6—C5	−1.0 (3)	C16—C17—C18—C19	0.2 (3)
N1—C1—C6—C5	177.45 (16)	C17—C18—C19—C20	−0.2 (3)
C2—C1—C6—C7	179.79 (16)	C18—C19—C20—C21	−0.1 (3)
N1—C1—C6—C7	−1.78 (19)	C19—C20—C21—C16	0.3 (3)
C4—C5—C6—C1	0.5 (3)	C17—C16—C21—C20	−0.4 (3)
C4—C5—C6—C7	179.5 (2)	S1—C16—C21—C20	179.02 (14)
C1—C6—C7—C8	0.1 (2)	C2—C1—N1—C8	−179.02 (17)
C5—C6—C7—C8	−179.0 (2)	C6—C1—N1—C8	2.74 (17)
C1—C6—C7—C22	176.8 (2)	C2—C1—N1—S1	−35.5 (2)
C5—C6—C7—C22	−2.2 (4)	C6—C1—N1—S1	146.23 (12)
C6—C7—C8—N1	1.6 (2)	C7—C8—N1—C1	−2.74 (19)
C22—C7—C8—N1	−175.0 (2)	C9—C8—N1—C1	−175.68 (16)
C6—C7—C8—C9	174.50 (17)	C7—C8—N1—S1	−146.25 (14)
C22—C7—C8—C9	−2.1 (3)	C9—C8—N1—S1	40.8 (2)
C7—C8—C9—O3	62.9 (3)	C1—N1—S1—O2	−178.64 (12)
N1—C8—C9—O3	−125.3 (2)	C8—N1—S1—O2	−40.07 (15)
C7—C8—C9—C10	−109.4 (2)	C1—N1—S1—O1	52.73 (14)
N1—C8—C9—C10	62.4 (3)	C8—N1—S1—O1	−168.70 (12)
O3—C9—C10—C11	−9.9 (3)	C1—N1—S1—C16	−63.40 (13)
C8—C9—C10—C11	162.15 (18)	C8—N1—S1—C16	75.17 (13)
O3—C9—C10—C15	171.9 (2)	C17—C16—S1—O2	33.31 (15)
C8—C9—C10—C15	−16.0 (3)	C21—C16—S1—O2	−146.06 (14)
C15—C10—C11—C12	0.0 (3)	C17—C16—S1—O1	166.94 (13)
C9—C10—C11—C12	−178.23 (18)	C21—C16—S1—O1	−12.44 (15)
C10—C11—C12—C13	−1.2 (3)	C17—C16—S1—N1	−79.03 (14)
C11—C12—C13—C14	1.0 (3)	C21—C16—S1—N1	101.60 (14)
C12—C13—C14—C15	0.4 (3)		

### Hydrogen-bond geometry (Å, °)

**Table:**

*D*—H···*A*	*D*—H	H···*A*	*D*···*A*	*D*—H···*A*
C2—H2···O1	0.93	2.45	3.025 (2)	120
C15—H15···N1	0.93	2.61	3.254 (2)	127
C21—H21···O1	0.93	2.53	2.904 (2)	104
C21—H21···O2^i^	0.93	2.29	3.127 (2)	149.

Symmetry codes:  (i) −*x*+1/2, *y*−1/2, −*z*+1/2.
